# P35 Heteroresistance to cefiderocol in clinical Gram-negative isolates

**DOI:** 10.1093/jacamr/dlad066.039

**Published:** 2023-06-14

**Authors:** Celeste Watson, Simran More, Sylvia Rofael, Timothy McHugh, Indran Balakrishnan

**Affiliations:** UCL Centre for Clinical Microbiology, University College London, London, UK; UCL Centre for Clinical Microbiology, University College London, London, UK; UCL Centre for Clinical Microbiology, University College London, London, UK; UCL Centre for Clinical Microbiology, University College London, London, UK; UCL Centre for Clinical Microbiology, University College London, London, UK; Microbiology Department, The Royal Free Hospital, London, UK

## Abstract

**Background:**

Heteroresistance is a phenotype in which a clinically susceptible bacterial isolate contains less susceptible subpopulations. Unexpectedly high levels of treatment failure for cefiderocol, a novel antibiotic designed to treat multidrug-resistant infections, have been attributed to this phenomenon.

**Objectives:**

To determine prevalence of cefiderocol heteroresistance amongst clinical isolates at a North London tertiary care centre and study the population dynamics upon exposure to the antibiotic.

**Methods:**

Gram-negative clinical isolates (*n* = 146) were screened for cefiderocol susceptibility by standard disc diffusion and Etest assays. Isolates were classified based on EUCAST breakpoints for cefiderocol susceptibility. Heteroresistance was confirmed by performing modified-population analysis profile (mPAP) assay in iron-depleted Mueller Hinton II agar. MIC values were determined by broth microdilution (BMD) assay as per EUCAST guidelines. The heteroresistant isolates and their corresponding less susceptible subpopulations were serially passaged in media with sub-inhibitory concentrations of cefiderocol as well as in cefiderocol-free medium. The changes in frequency and/or susceptibility in the heteroresistant subpopulation were detected using mPAP.

**Results:**

Eleven heteroresistant (7.53%) and twenty-two resistant (15.06%) isolates were identified. Cefiderocol susceptibility of the heteroresistant isolates after five passages in cefiderocol-free medium partially reverted to that of the parent population. However, this phenomenon was not observed in the cohort that was first passaged in cefiderocol-containing medium before passages in cefiderocol-free medium. When heteroresistant populations were passaged in cefiderocol-containing media at 0.25× MIC values, no MIC changes were observed after five serial passages; however, the proportion of less susceptible subpopulations increased by 1.2 log_10_ in one out of five isolates. Nevertheless, when the antibiotic pressure was removed by passaging in cefiderocol-free media for a further five serial passages, the MIC and/or frequency of the less susceptible colonies within the heteroresistant population further increased in 80% of the passaged isolates. In one isolate, the MIC of the heteroresistant sub-population increased by >32-fold while the frequency of the heteroresistant sub-population increased by 1.0 log_10_ in another.

**Conclusions:**

The prevalence of heteroresistance to cefiderocol was around 7% amongst clinical isolates. The heteroresistant phenotypes were unstable unless exposed to cefiderocol. Such exposure may also increase the MIC and/or frequency of less susceptible cells within the heteroresistant population. This amplification can continue even after removal of antibiotic pressure. Developing methods for detecting heteroresistance to cefiderocol in clinical laboratories is essential. Clinicians need to take this phenomenon into account when planning treatment regimens to optimize the effectiveness of this invaluable addition to the antimicrobial armamentarium.

